# Allergenicity Assessment of Precision Fermentation-Derived Food Proteins

**DOI:** 10.3390/foods15173153

**Published:** 2026-09-05

**Authors:** Jun-Hyeok Ham, Heewon Jung, Chaemin Hong, Hae-Yeong Kim

**Affiliations:** Department of Food Science and Biotechnology, Kyung Hee University, Yongin 17104, Republic of Korea; wns5126@khu.ac.kr (J.-H.H.); jhwon11n14@khu.ac.kr (H.J.); kyungofficial01@khu.ac.kr (C.H.)

**Keywords:** precision fermentation, novel food, allergenicity assessment, food safety, food processing

## Abstract

Precision fermentation (PF) yields single, well-characterized recombinant proteins, such as dairy and egg white proteins, at an industrial scale, and several products have reached the market. Numerous PF-derived proteins, including ovomucoid and β-lactoglobulin, are known allergens designed as exact structural copies of their native homologs; hence, assessments center on equivalence rather than novel hazards. Therefore, the current tiered scheme of allergen database comparison, digestibility testing, and serum immunoglobulin E binding assays is applicable. Nevertheless, residual host cell proteins, altered host-dependent enzymatic glycosylation, and process-related impurities render PF-derived products imperfect replicas, and thermal and high-pressure processing, enzymatic hydrolysis, and non-enzymatic glycation can modify linear or conformational epitopes, thereby reducing IgE binding or generating neoepitopes. Because these effects depend on structural attributes acquired during production, including folding stability, disulfide bonding, glycosylation, and proteolytic resistance, PF-derived proteins and native proteins may respond differently to identical processing. This review explores the molecular attributes acquired at each stage of PF production and the epitope changes originating during processing, reevaluates the current assessment scheme, and outlines considerations for a premarket safety framework reflecting realistic processing and consumption conditions.

## 1. Introduction

The global protein demand is increasing with population growth and dietary changes; however, livestock-based production faces structural restrictions in terms of greenhouse gas emissions and land and water use [1]. Consequently, researchers have proposed alternatives, including plant proteins, insect proteins, microalgae, and cultured meat [2]. In this context, precision fermentation (PF) is a technique that uses genetically engineered microorganisms to directly produce specific animal proteins [3]. Unlike traditional fermentation, which exploits the overall metabolic output of microorganisms, in PF, the gene encoding the target protein is introduced into a host to produce a defined molecule at high purity. PF is also distinct from biomass fermentation, in which edible microorganisms are themselves used as a source of edible biomass and functional ingredients rather than as hosts for a defined recombinant product [4]. This technology has already moved beyond proof of concept, with recombinant β-lactoglobulin and ovomucoid having received regulatory approval in the United States, and products formulated with them having entered the market [5]. As of 1 September 2026, eleven notices received “no questions” letters from the US Food and Drug Administration (FDA) under the generally recognized as safe (GRAS) notification program, all for recombinant milk or egg proteins (Table 1).

As PF-derived proteins enter the food supply chain, it is necessary to establish their safety [6]. In particular, the primary targets of PF are milk and egg white proteins, the major allergens subject to mandatory labeling in most countries [7]. For protein sources with allergenicity that remains poorly characterized, such as insects and microalgae, assessment is aimed at identifying unknown hazards [8]. In contrast, PF deliberately reproduces proteins already established as allergens; hence, the question differs in type. It is not a question of whether the protein triggers an allergy, but whether the recombinant product behaves as its native homolog does. This equivalence is not self-evident because host-dependent post-translational modifications (PTMs), N-terminal heterogeneity originating from incomplete signal peptide cleavage, and host-derived material persist after purification, causing the final product to deviate subtly from the native protein [9].

Moreover, PF-derived proteins are not consumed in purified form but are formulated as functional ingredients in dairy and egg white alternatives and thus undergo processing [10]. The fact that such food processing alters protein allergenicity has been widely documented for established allergens, and the direction is not uniform, with studies showing that IgE reactivity may decrease when denaturation and aggregation destroy epitopes or increase when hidden epitopes are exposed or neoepitopes are formed [11,12]. Critically, the attributes regulating these responses to processing, viz., tertiary structure stability, disulfide bonding, glycosylation, and proteolytic resistance, are the very attributes established during production [13]. Hence, PF-derived proteins may respond differently from their native counterparts under identical processing conditions, and their allergenicity is determined not by the target sequence alone but by the entire history of production and processing.

Processing itself is not unique to PF-derived proteins, because their native counterparts are likewise formulated and heated. What differs is the substrate entering processing and the matrix in which processing occurs. The production-established attributes described above arise from biosynthetic machinery that differs between the two, as PF hosts assemble them through microbial secretory and modification systems rather than the bovine mammary gland or the avian oviduct. The two proteins consequently do not enter processing in the same structural state. The matrix differs as well. Native β-lactoglobulin is heated among caseins and lactose [14], whereas the same protein enters a dairy alternative as a purified isolate and may be heated without micellar casein and with different reducing sugars. This alters both the partners available for heat-induced aggregation and the resulting glycation profile [15,16], that is, the non-enzymatic attachment of reducing sugars during processing and storage, which is distinct from the glycosylation added enzymatically within the producing cell. Processing effects characterized for native milk and egg proteins therefore cannot be assumed to transfer to their PF-derived equivalents. Direct comparisons under matched conditions remain scarce, and this gap leaves a scientific uncertainty that premarket assessment must resolve.

The current allergenicity assessment framework was established for newly expressed proteins in genetically modified organism (GMO) foods and later extended across novel foods. It follows a tiered structure, beginning with a literature review and sequence homology analysis and proceeding to digestibility, serum IgE binding, and clinical challenge testing [17]. However, this framework was designed to detect hazards in proteins of unknown allergenicity, whereas PF-derived proteins reproduce known allergens and also carry a dual uncertainty from production and processing; hence, the direct applicability of this framework requires separate consideration [18,19].

Previous reviews of PF-derived proteins have focused mainly on production technology and techno-functional properties, and allergenicity has rarely been examined in relation to the production and processing steps that shape it. This review therefore focuses on recombinant milk and egg white proteins of animal origin, which are the principal commercial targets of PF and are themselves regulated allergens, with three objectives: (i) to describe the molecular attributes acquired at each stage of PF production, from host selection to downstream processing, and identify where these deviate from the native homolog; (ii) to examine how heat treatment, high-pressure processing, enzymatic hydrolysis, and glycation can modify IgE-binding epitopes, and what these established effects imply for PF-derived proteins that may differ in the attributes governing them; and (iii) to reexamine each tier of the current assessment framework, from the literature review and sequence homology analysis to digestibility testing, serum IgE binding, and clinical challenge, and determine what each tier can establish once assessment centers on equivalence to a known allergen rather than on the identification of an unknown hazard. Drawing on these three lines of evidence, this review identifies the scientific and regulatory issues that must be resolved before the allergenicity of PF-derived proteins can be assessed with confidence.

## 2. Production of PF-Derived Proteins

**Figure 1:**
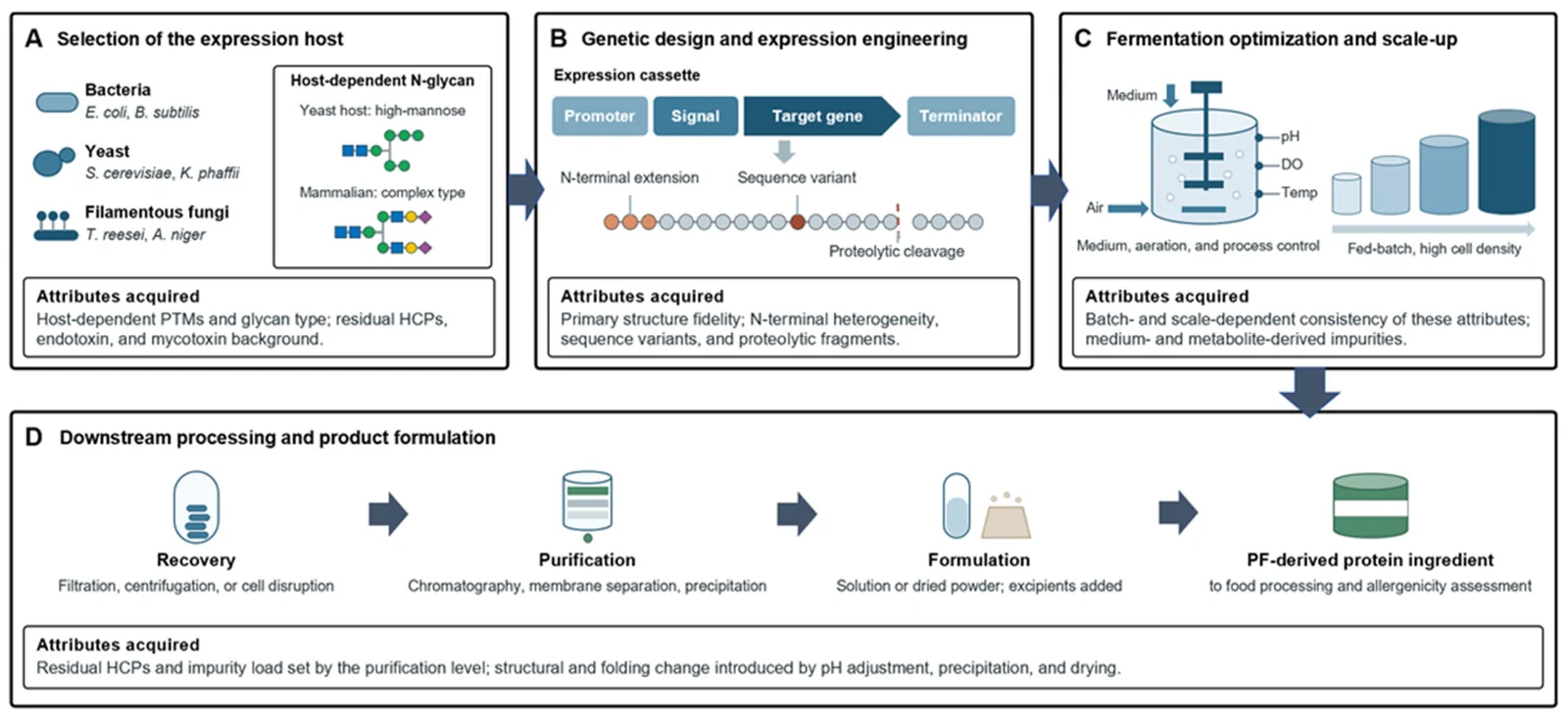
Production of precision fermentation (PF)-derived proteins and the molecular attributes acquired at each stage. Upstream processing establishes the biological basis for expression through (**A**) selection of the expression host; (**B**) genetic design and expression engineering; and (**C**) fermentation optimization and scale-up, after which (**D**) downstream processing recovers and purifies the expressed protein and converts it into a product form.

The production of PF-derived proteins spans upstream and downstream processing, from host selection to final formulation. Upstream processing establishes the biological basis for expression, covering host selection, genetic design and expression engineering, and fermentation optimization and scale-up. Downstream processing then recovers and purifies the expressed protein and converts it into a product form (Figure 1). The choices made at each stage determine not only yield and purity but also the molecular attributes relevant to allergenicity assessment, including host-dependent PTMs, residual host cell proteins (HCPs), and process-related impurities. This section outlines each stage with emphasis on the attributes it introduces.

### 2.1. Selection of the Expression Host

Host selection is the starting point of the PF process and a vital determinant of the properties of the final protein. It is based on the physicochemical characteristics of the target protein and on the genetic tractability and metabolic versatility of the host [3,20]. Food-grade hosts must additionally be free of pathogenicity and toxin production and be genetically stable [21]. Bacterial platforms, especially *Escherichia coli* and *Bacillus subtilis*, provide high yields at low cost but lack eukaryotic glycosylation machinery and consequently produce nonglycosylated proteins, with residual endotoxin remaining a concern for Gram-negative hosts such as *E. coli* [22]. Yeast platforms are the primary hosts for food protein production due to their PTM capacity and history of safe use. Representative examples are *Saccharomyces cerevisiae*, which holds GRAS status, and *Komagataella phaffii*, which is widely used in the production of β-lactoglobulin and ovalbumin [3]. Filamentous fungi such as *Trichoderma reesei* and *Aspergillus niger* secrete proteins efficiently but retain the capacity to produce mycotoxins [22].

The primary association between host selection and allergenicity is that PTMs, particularly glycosylation, vary with the host. Therefore, a protein with an identical amino acid sequence may differ in structural and functional behavior depending on the production host [22]. For instance, yeast N-glycans are of the high-mannose type rather than the complex type found in mammals [23]. Such host-dependent differences in glycosylation could in principle alter IgE binding or generate neoepitopes [24], although there is limited quantitative evidence for food proteins.

The host further defines the impurities and hazards that may persist in the final product. Residual HCPs, host-derived polysaccharides, endotoxins, and mycotoxins originate from host choice, and the target protein is purified against this background. Hence, host selection establishes the immunochemical baseline of the final product not through the target sequence but through the surrounding PTMs and impurity background, making it the starting condition for subsequent assessment.

Host choice thus acts on allergenicity through two distinct routes: the direct presence of host-derived material in the product, and the imprint the host leaves on the molecular attributes of the target protein itself. Evidence on how these routes differ between hosts remains scarce, and a systematic comparison is needed before host selection can be informed by allergenic potential (Table 2).

### 2.2. Genetic Design and Expression Engineering

Host selection is followed by genetic design and expression engineering. The expression cassette combines a codon-optimized target gene with a promoter, secretion signal, and terminator. These elements, together with vector choice and copy number, are tuned for transcription, translation, and secretion efficiency [23]. In *K. phaffii*, the methanol-inducible *AOX1* promoter and the α-mating factor signal sequence from *S. cerevisiae* are widely used; however, signal sequence efficiency varies with the protein, and hence, endogenous and designed signal sequences serve as alternatives [25]. At the pathway level, genome editing and metabolic engineering increase flux and yield toward the target while suppressing the generation of by-products [26], and host pathways are adjusted in parallel when cofactors or specific PTMs are required.

These design choices bear directly on allergenicity because they define the primary structure of the final protein. The general goal is a sequence identical to the native homolog; however, deviations arise. Incomplete cleavage of the secretion leader produces N-terminal heterogeneity, as demonstrated for bovine β-lactoglobulin secreted from *P. pastoris*, in which N-terminal sequencing showed that the Glu-Ala spacer repeats of the α-mating factor prepro-leader remained attached to the mature protein; notably, the extended and native proteins were indistinguishable by circular dichroism, denaturant-induced unfolding, crystallization and NMR, indicating that conformational comparison alone does not detect such deviations [27]. Comparable heterogeneity has been reported for hen ovalbumin expressed in *P. pastoris*, which was recovered as two molecular species and lacked the N-terminal acetylation of the native protein [28]. Variants introduced to improve functionality alter the sequence itself, and partial proteolysis by host proteases during secretion yields fragmented forms [29]. Therefore, protease-deficient strains contribute to both structural homogeneity and yield. These deviations are relevant to allergenicity because the N-terminal region of β-lactoglobulin lies within the part of the molecule that carries sequential IgE-binding epitopes [30]. Whether leader-derived extensions change IgE recognition has not been tested, as no study has compared extended and correctly processed proteoforms using patient sera. Any such deviation can in principle generate neoepitopes, the same concern that has long been applied to newly expressed proteins in GMO foods [31]. Thus, expression engineering shapes immunochemical properties in both directions, deliberately through glycoengineering and inadvertently through these deviations, making the verification of sequence fidelity and expression homogeneity vital to product characterization.

### 2.3. Fermentation Optimization and Scale-Up

After strain construction, cultivation determines productivity through medium composition and process control. The medium, including carbon and nitrogen sources and trace elements, is designed to fulfill the requirements of the host and the introduced pathway [32]. Bioreactor scale and configuration, agitation and aeration, pH, and temperature control cell growth, metabolic flux, and target protein accumulation, and the established conditions are scaled up through fed-batch and high-cell-density cultivation [33,34].

Cultivation is related to allergenicity not through a specific molecular mechanism but through the consistency of product quality. Product quality attributes are established by strain design; however, whether they are realized uniformly depends on dissolved oxygen, pH, temperature, induction conditions, and culture stage [35]. In large bioreactors, spatial gradients in oxygen, pH, and substrate concentration impose local stress and generate population heterogeneity that are absent at the laboratory scale, and medium components and metabolic by-products form an impurity background carried into downstream processing [36]. Two consequences bear on the attributes relevant to allergenicity. Oxygen limitation and pH excursions alter flux through the secretory pathway, so the extent and uniformity of glycan processing may vary between batches even for an unchanged strain. Vacuolar proteases also accumulate in the broth as cultivation proceeds, at levels that vary with culture pH and carbon source, and the secreted product is then exposed to proteolysis that can trim termini and generate fragmented proteoforms while lysed cells add to the HCP background [37]. Neither consequence has been shown to alter allergenicity directly, but both act on the very attributes on which equivalence to the native homolog is judged. Hence, a fundamental consideration at this stage is whether immunochemically relevant attributes are reproduced across batches and scales.

The composition of the medium is itself relevant to allergenicity. Complex nutrients such as yeast extract, peptones and corn steep liquor remain widely used despite their ill-defined composition and batch-to-batch variability [38], and peptone is an enzymatic digest of animal protein [39]. Food enzyme assessment accounts for this. The European Food Safety Authority (EFSA) guidance lists proteinaceous material with known allergenic properties in the fermentation medium among the elements considered, and accepts an argument based on lack of transfer only where the food enzyme total organic solids are absent from the final food [40]. In a recent assessment of an enzyme produced with *T. reesei*, a host also used for PF, allergenic materials listed under Regulation (EU) No 1169/2011 were present in the culture medium, and the panel concluded that residual allergenic proteins remained in the enzyme [41]. PF-derived proteins cannot invoke the exemption, because they are constituents of the final food rather than processing aids removed during manufacture. Chemically defined media contain no protein hydrolysates and offer greater batch-to-batch consistency [39]. Where complex components are retained, the raw materials used and the extent of their removal during downstream processing require documentation [40].

### 2.4. Downstream Processing and Product Formulation

Downstream processing recovers and purifies the target protein from the culture broth and converts it into a final formulation. Secreted proteins are separated from cells by filtration or centrifugation, whereas intracellular proteins are released by cell disruption [3]. The recovered protein is then purified by chromatography, membrane separation, or selective precipitation, and the level of purification, established by the intended application and required purity, determines how much host-derived material remains in the product [34]. The purified protein is maintained in solution or dried into powder, and excipients are added as required to complete the formulation.

These options differ in resolving power and in cost, and food protein production is constrained by economic feasibility. Chromatography delivers the highest purity but is designed for high-purity, high-value products, and bulk food proteins require alternative downstream approaches [42]; dairy proteins already demand more intensive downstream processing than enzymes or pigments [43], and membrane separation and selective precipitation are the practical alternatives at the food scale despite their lower resolution. The purity target also differs from that of pharmaceutical proteins, for which residual host cell proteins are monitored as a process-related impurity and their acceptable levels are reviewed case by case [44]. No comparable specification applies to food proteins, which are consumed in gram quantities as ingredients and purified only to the level the intended use requires. PF-derived food proteins therefore carry a larger residual burden of host-derived material than a pharmaceutical protein of the same sequence, and the purification route determines not only cost and yield but also the allergenic risk contributed by material other than the target protein itself.

The physicochemical treatments involved in downstream processing and formulation can also modify the structure of the target protein itself. Chemical stress during pH adjustment, precipitation, and drying can alter native structure and folding, and drying almost always compromises product quality even without denaturation [42]. Consequently, downstream processing is the point at which processing-induced structural change begins, before the product enters food processing. Altogether, downstream processing determines the residual impurity background and modifies the structure; hence, the allergenicity of the product is completely captured only when the impurity profile and the structure are analyzed together in the final formulated state.

## 3. Processing-Induced Changes in the Allergenicity of Food Proteins

Food processing alters allergenicity by modifying IgE-binding epitopes. Thermal and nonthermal treatments destroy linear and conformational epitopes through denaturation, aggregation, and hydrolysis or expose hidden epitopes and form neoepitopes through covalent modification [45]. The direction is not uniformly predictable, as it relies on the combination of allergen, processing conditions, and food matrix [13]. These responses are controlled by folding stability, disulfide bonding, glycosylation, and proteolytic resistance, the same attributes that determine allergenicity itself.

**Figure 2:**
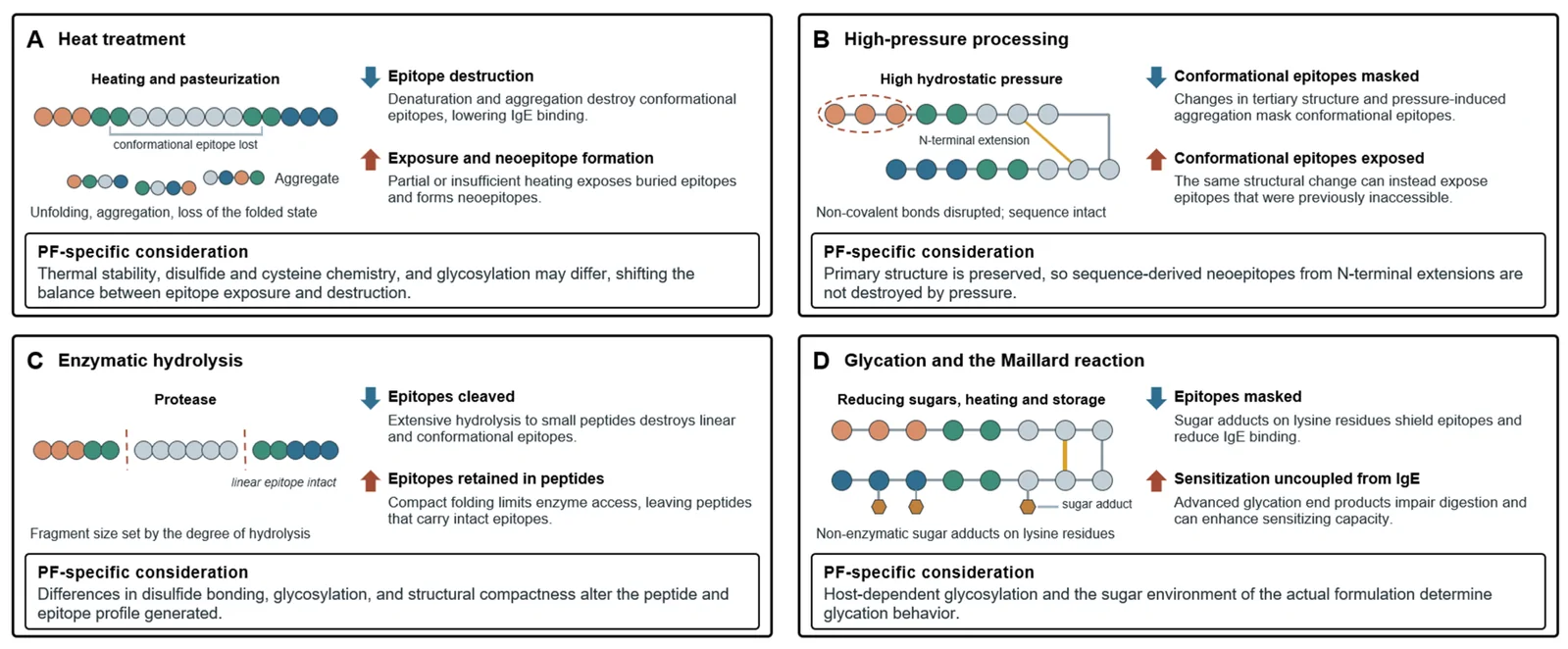
Processing-induced changes in the allergenicity of PF-derived food proteins. Each panel shows how one treatment alters the protein and its IgE-binding epitopes, namely (**A**) heat treatment, (**B**) high-pressure processing, (**C**) enzymatic hydrolysis, and (**D**) glycation and the Maillard reaction, with downward and upward arrows marking the reported decrease and increase in IgE binding. In the schematics, gray denotes unmodified residues, blue a linear epitope, green the two segments of a conformational epitope, orange an N-terminal extension, yellow a disulfide bond, and the hexagons in (**D**) non-enzymatic sugar adducts.

PF-derived proteins are formulated as functional ingredients in dairy and egg white alternatives, where they undergo heating, pasteurization and high-pressure processing (HPP), enzymatic hydrolysis, and non-enzymatic glycation during storage (Figure 2). These treatments modify epitopes through distinct mechanisms, viz., denaturation and aggregation, disruption of noncovalent interactions, cleavage of covalent bonds, and formation of new covalent adducts. PF-derived proteins may differ from their native homologs in the attributes controlling these reactions [9,46]. Host-dependent glycosylation has been directly demonstrated: β-lactoglobulin and ovalbumin produced in *T. reesei* were N-glycosylated, predominantly with Man5 [29], and N-glycans were likewise detected on β-lactoglobulin in a commercial PF whey ingredient [47]. Other proposed deviations differ in how well they are established. N-terminal heterogeneity arising from incomplete leader cleavage has been documented in individual yeast-expressed food proteins, but its prevalence across PF products and its consequences for processing behavior have not been systematically characterized. Non-native disulfide pairing and altered proteolytic susceptibility remain mechanistically plausible but have not been demonstrated in food proteins produced by the yeast and fungal hosts used for commercial PF. These deviations may consequently lead to different responses to identical processing. Because of the scarcity of direct data, each treatment is investigated against its established effects on known allergens as a baseline.

### 3.1. Heat Treatment

Heat treatment reduces allergenicity by destroying epitopes through denaturation, aggregation, and the Maillard reaction or increases it by exposing hidden epitopes and forming neoepitopes, with the direction depending on the allergen, processing conditions, and matrix [13,45]. For instance, β-lactoglobulin, a primary PF target, is stabilized by two intramolecular disulfide bonds and begins to change conformation at >65 °C. Its IgE-binding capacity decreases overall as conformational epitopes are destroyed [48], whereas milder heating transiently increases antigenicity by unfolding the structure and exposing buried epitopes [49]. In contrast, casein is dominated by linear epitopes and is heat-stable, largely retaining its IgE reactivity after heating [50].

Remarkably, numerous studies have reported that heat treatment instead increases allergenicity. For instance, in egg white, insufficient heating increased IgE binding compared with the raw protein through neoepitope formation [51], and a recent study reported that egg white allergenicity varies with the cooking method [52]. Nevertheless, such assessments generally depend on IgE binding. For instance, studies have shown that roasted Ara h 1 and heated pea proteins exhibited reduced IgE binding, whereas mast cell and basophil reactivity was maintained or even increased [53,54], indicating that IgE binding alone cannot determine clinical allergenicity.

PF-derived proteins could differ from their native homologs in thermal stability, disulfide and cysteine chemistry, and glycosylation, and the balance between epitope exposure and destruction could therefore shift under identical processing conditions. Direct evidence remains sparse. In a reported side-by-side comparison of a PF-derived protein and its native counterpart, the heat-induced gel strength of *T. reesei*-produced ovalbumin was somewhat lower than that of hen ovalbumin, although the authors attributed this to partial degradation or residual host proteins rather than to an intrinsic difference in heat stability [29]. For disulfide chemistry, recombinantly produced β-lactoglobulin has been shown to depend on correct disulfide connectivity for proper folding, and minor differently folded isomers were detected even in the wild-type recombinant protein; the authors of that study noted that microbial production may not fully reproduce the in vivo folding process of β-lactoglobulin in ruminants, although their work used an *E. coli* host rather than the yeast and fungal systems used for commercial PF [55]. Whether comparable folding deviations occur in yeast- or fungus-derived food proteins, and whether they alter IgE-binding profiles after identical heat treatment, has not been examined. As no validated method for predicting or screening neoepitope formation is yet available [17,18], both reduction and potential increases in allergenicity require empirical verification for PF-derived proteins.

### 3.2. High-Pressure Processing

HPP disrupts noncovalent interactions such as hydrogen bonding and hydrophobic and electrostatic interactions, thereby altering secondary and tertiary structures but leaving the primary structure intact [12,56]. Unlike heat treatment, which can modify both linear and conformational epitopes, HPP leaves linear epitopes unchanged and alters allergenicity primarily through the exposure or masking of conformational epitopes and through pressure-induced aggregation [45]. Nevertheless, the outcome varies with the protein. For β-lactoglobulin, a primary PF target, IgE binding was lowest at 200 MPa and highest at 400 MPa, whereas the primary and secondary structures remained stable, and only the tertiary structure changed [57]. In ovalbumin, antigenicity decreased instead through pressure-accelerated pepsin hydrolysis, and even then residual IgG and IgE binding persisted [58]. Therefore, conformational stability and susceptibility to proteolysis regulate both the direction and magnitude of the response to pressure.

PF-derived proteins may differ from native proteins in the attributes that control these responses [9]. Because HPP preserves the primary structure, any sequence-derived epitope arising from an N-terminal extension would, in principle, persist after pressure treatment, although aggregation could restrict its accessibility. This remains a theoretical expectation: whether such extensions constitute IgE-binding epitopes, and how they behave under pressure, has not been experimentally examined. Hence, the response of PF-derived proteins to HPP requires product-specific verification based on conformational stability and sequence deviation.

### 3.3. Enzymatic Hydrolysis

Enzymatic hydrolysis cleaves peptide bonds and thereby destroys both linear and conformational epitopes; it is the most established approach for generating hypoallergenic products such as infant formula [59]. For instance, in milk protein concentrate, extensive hydrolysis yielded peptides <3 kDa with markedly reduced allergenicity compared with that of the intact protein [60]. The effect strongly depends on the enzyme and the degree of hydrolysis, with partial hydrolysates retaining larger peptides than extensive hydrolysates, and hydrolysis alone generally leaves residual allergenicity [59].

The efficiency of hydrolysis depends on how susceptible a protein is to proteolysis, which is controlled by disulfide bonding, glycosylation, and structural compactness [13]. Compactly folded allergens restrict enzyme access, and single-enzyme hydrolysis often leaves peptides that retain intact epitopes [61]. PF-derived proteins may differ from native proteins in precisely these attributes and may consequently be hydrolyzed to a different extent under identical treatment, yielding different peptide and epitope profiles whose immunoreactivity cannot be assumed [11]. Comparative in vitro pepsin digestion of a PF-derived protein alongside its native counterparts has so far been reported only for human lactoferrin, the native form of which is not a major food allergen [62]. To our knowledge, no equivalent comparison has been published for the PF-derived milk and egg allergens that are the focus of this review, and the expectation of divergent behavior therefore remains inferential. Because proteolytic resistance is also applied in allergenicity assessment as pepsin digestion stability, the digestive behavior of PF-derived products requires product-specific verification.

### 3.4. Glycation and the Maillard Reaction

Glycation is a component of the Maillard reaction wherein reducing sugars bind none-nzymatically to the ε-amino groups of lysine and to the N-terminus, forming Amadori products and advanced glycation end products (AGEs), and it proceeds spontaneously during heating, sterilization, and storage [63]. It is mechanistically distinct from glycosylation, which is added enzymatically within the cell [9]. Glycation can mask epitopes and reduce IgE binding or increase it by exposing hidden epitopes and generating new ones, and the direction is inconsistent because it depends on the sugar, the extent of glycation, the protein, and the food matrix [63].

In β-lactoglobulin, a primary PF target, glycation with various sugars generally reduces IgE binding through epitope masking [64]. However, reduced IgE binding does not establish reduced allergenicity. Glycated β-lactoglobulin did not attenuate the ex vivo immune response of sensitized lymphocytes but instead improved it [65], and AGEs formed in peanut Ara h 1 and Ara h 3 during roasting impaired digestion and increased sensitizing capacity through the AGE receptor [63]. Therefore, glycation can decouple IgE binding from in vivo sensitization. PF-derived products carry host-dependent glycosylation that differs from the native pattern; this has been demonstrated both for laboratory-produced β-lactoglobulin and ovalbumin from *T. reesei*, which carried Man5 glycans predominantly [29], and for a commercial PF whey ingredient, in which high-mannose N-glycans were detected on β-lactoglobulin, whereas bovine whey carried complex and hybrid structures with sialic acid [47]. Because glycation depends on the availability of amino groups and on the sugar environment, these differences could alter glycation behavior, although no study has yet compared the glycation products or the resulting immunoreactivity of PF-derived and native proteins.

## 4. Allergenicity Assessment of PF-Derived Proteins

**Figure 3:**
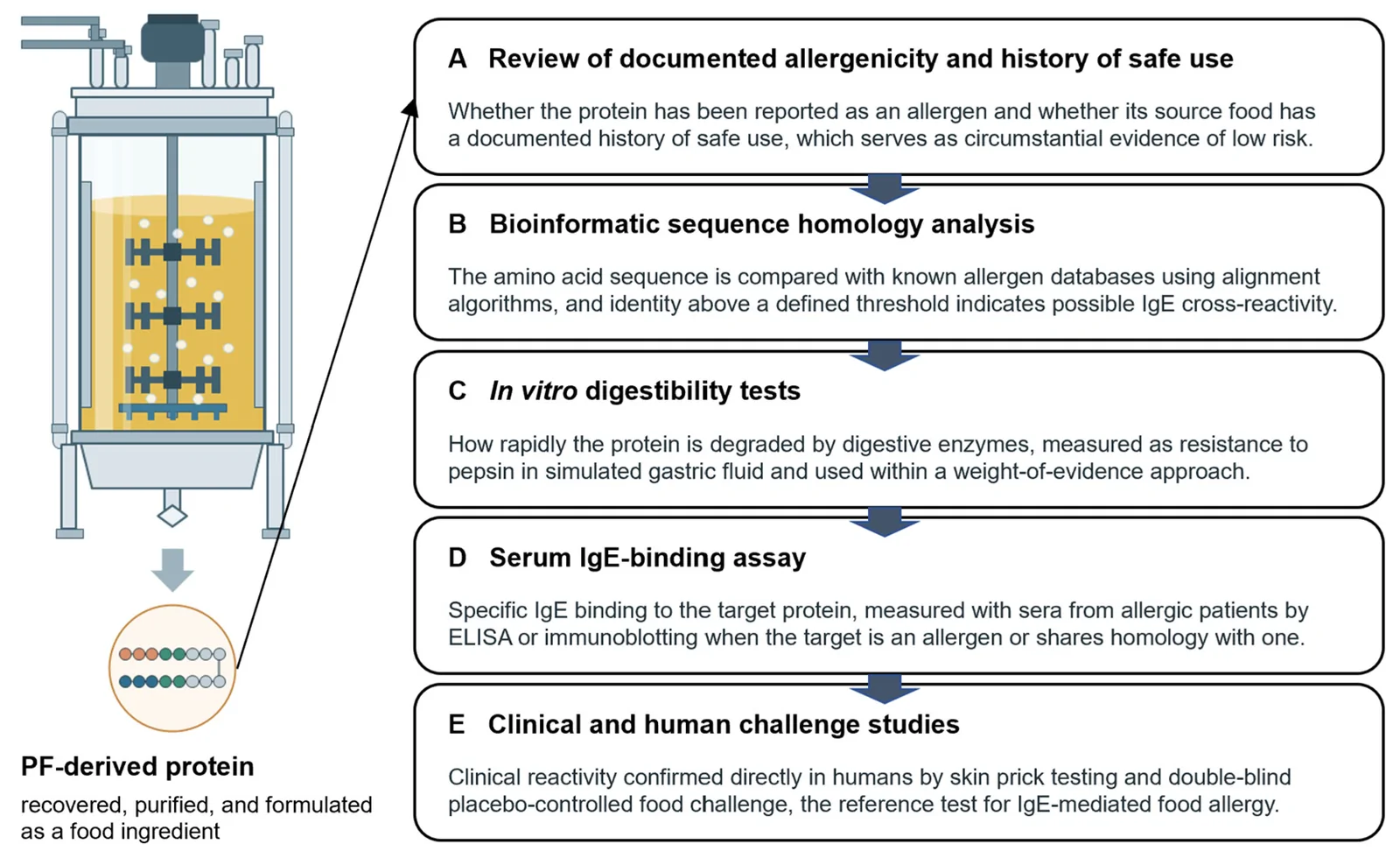
Tiered allergenicity assessment of PF-derived proteins. The recombinant protein, recovered and formulated as a food ingredient, is evaluated sequentially through five tiers, namely (A) review of documented allergenicity and history of safe use, (B) bioinformatic sequence homology analysis, (C) in vitro digestibility tests, (D) serum IgE-binding assay, and (E) clinical and human challenge studies.

Allergenicity assessment of PF-derived proteins applies the single-protein framework established for GMO foods and later extended to novel foods, an approach that fits directly because PF-derived products are expressed as single, well-characterized recombinant proteins. Assessment is tiered rather than based on a single test; thus, evidence is accumulated sequentially, including review of documented allergenicity and history of safe use, sequence homology analysis against known allergens, digestibility testing, serum IgE-binding assays, and, where warranted, clinical studies (Figure 3). Each tier confirms or excludes concerns raised by the preceding one, and evaluation stops once lower-tier evidence is conclusive. The following subsections address these five tiers in turn, with considerations specific to PF-derived proteins.

### 4.1. Review of Documented Allergenicity and History of Safe Use

The first tier utilizes the existing literature to establish two lines of evidence, i.e., whether the protein has been reported as an allergen and whether its source food has a documented history of safe use [66]. A history of safe use serves as circumstantial evidence of low risk, because widespread consumption over a long period without significant allergic problems indicates tolerance [67]. For foods such as milk and eggs, which contain well-characterized allergens despite a long consumption history, that history both supports safety and confirms the presence of the allergen.

For PF-derived products, both lines of evidence derive from the native homolog. β-Lactoglobulin and ovalbumin are the major allergens of milk and egg white, with allergenicity and clinical profiles already characterized [68], as well as the consumption history of the source foods. Nevertheless, this evidence pertains to the native protein, and the recombinant protein has no independent history of consumption. Hence, the native profile provides a robust baseline; however, whether structural deviations preserve this profile needs to be confirmed at subsequent tiers.

### 4.2. Bioinformatic Sequence Homology Analysis

The second tier compares the amino acid sequence of the target protein against known allergen databases to predict the potential for IgE cross-reactivity [69]. Databases such as AllergenOnline, COMPARE, and WHO/IUIS are searched using FASTA or BLAST algorithms, and a sequence identity of >35% over a window of 80 amino acids is considered, indicating possible cross-reactivity [17,67].

For PF-derived products, the informative content of this analysis lies in regions where the recombinant sequence departs from the native one [55]. Because the target itself is essentially identical to the database entry, the search returns a trivial complete match. Whether N-terminal extensions or introduced sequence variants generate new motifs matching other allergens or alter known epitopes of the native protein, they must be investigated.

### 4.3. In Vitro Digestibility Tests

The third tier evaluates how rapidly the target protein is degraded by digestive enzymes, typically by measuring the resistance to pepsin in simulated gastric fluid. The rationale is that a protein must survive digestion and reach the intestine in an immunologically active form to cause sensitization or an allergic reaction, and consequently, digestion-resistant proteins have been considered more allergenic [70]. This correlation is not absolute; rapidly digested allergens exist, small peptides may retain epitopes after digestion [71], and no established methodology is available for characterizing such stable fragments. Accordingly, recent evidence does not support the classical pepsin resistance test as a good predictor of allergenic potential, and it is used as supporting information within a weight-of-evidence approach [72].

For PF-derived products, this test focuses on whether structural deviations have altered the digestive behavior compared with the native protein. Digestive resistance depends on disulfide bonding, glycosylation, and structural compactness [13], attributes in which PF-derived products may differ; hence, the rate of digestion and the resistant peptides generated may also differ. Such differences would indicate altered epitope exposure and persistence during digestion and, if observed, would require further verification at the IgE level. In practice, the test is applied comparatively rather than as a pass/fail criterion, because the native form of a PF target is itself already known to resist digestion. In the only published application to a PF-derived food protein, recombinant human lactoferrin was digested in simulated gastric fluid alongside human milk and bovine lactoferrin, and equivalence was claimed on the basis that its degradation profile matched that of the human milk protein rather than on the absolute rate of degradation [62]. Two operational requirements follow for milk and egg proteins. The native comparator must be digested in the same run, since inter-assay variation in pepsin activity and pH is a principal source of discordance between laboratories [72]. And because the deviations of interest are localized—a leader-derived N-terminal extension, an altered disulfide, a host-type glycan—the readout must resolve which peptides survive rather than only whether the intact band disappears [71].

### 4.4. Serum IgE-Binding Assay

The fourth tier directly measures specific IgE binding to the target protein using sera obtained from allergic patients and is performed when the target is an allergen or shares homology with a known allergen. IgE binding is typically determined by an enzyme-linked immunosorbent assay or immunoblotting and is divided into specific serum screening, which uses sera obtained from patients sensitized to the source or to related allergens, and targeted serum screening, which determines cross-reactivity using sera containing a wide range of IgE [73].

For PF-derived products, this assay can serve as the point at which the immunological consequences of structural deviation, only indirectly inferred at the sequence and digestibility tiers, are evaluated directly. Nevertheless, IgE binding indicates changes in epitope recognition rather than clinical reactivity; hence, a difference in binding cannot be considered a change in clinical allergenicity [74].

A practical difficulty specific to this product class is that a positive result is expected by design. Because PF-derived milk and egg proteins are deliberate copies of established allergens, IgE binding by sera from allergic individuals is the anticipated outcome and is not in itself a safety signal; what the assay must resolve is a difference in binding magnitude or epitope pattern relative to the native protein assayed in parallel. This comparative format has been applied to recombinant forms of these allergens, with results that diverge according to which attribute was altered. Recombinant and native ovomucoid resulted in identical IgE and IgG binding by ELISA, even though circular dichroism showed the recombinant protein to contain more α-helix and less β-structures [75], whereas a variant in which the cysteine bridges were disrupted showed clearly reduced IgE reactivity [76]. These outcomes follow the epitope-type dependence described for other allergens in Section 3.1: ovomucoid is dominated by sequential epitopes, four of which are recognized specifically by patients with persistent egg allergy [77], so a redistribution of the secondary structure leaves recognition largely intact, whereas loss of the disulfide architecture maintaining the domain fold does not. What determines the immunological outcome is therefore which attribute has changed, not whether the protein differs structurally in some measurable respect. Notably, the first of these epitopes begins at the mature N-terminus, the position at which α-factor-derived extensions are appended. Both studies used *E. coli*-expressed protein rather than PF material, and no PF-derived milk or egg protein has yet been assayed against its native counterpart in this format.

Two limitations bear on how such a comparison should be read. Because the sera were derived from patients sensitized to the native protein, altered conformational epitopes may lower binding to the recombinant target without a corresponding loss of clinical reactivity, giving a false-negative result. A further limitation is structural rather than technical. IgE-binding assays, of which ELISA is the most widely used, are defined by the antigen immobilized and by the IgE repertoire of the sera used. They therefore indicate the presence or absence of sensitization to a known protein rather than clinical allergy, and cannot identify whether a novel epitope is capable of sensitizing a previously nonallergic individual [78]. Allergenicity is therefore difficult to judge from IgE binding alone, and the result should be read alongside functional assays such as the basophil activation test within a weight-of-evidence approach.

### 4.5. Clinical and Human Challenge Studies

The final tier confirms clinical reactivity directly in humans through skin prick testing and an oral food challenge. In particular, the double-blind placebo-controlled food challenge is the gold standard for diagnosing IgE-mediated food allergy and the reference test for investigating new diagnostic and therapeutic approaches [79,80]. This tier occupies the highest position because sensitization and IgE binding do not necessarily correspond to clinical reactivity, and only a challenge can confirm whether a protein actually triggers clinical symptoms. Nevertheless, it carries a risk of severe allergic reactions and is resource-intensive; it is therefore performed under strict supervision only when significant concerns have been raised at lower tiers [81].

### 4.6. Allergenicity Assessment of a PF-Derived Protein in Practice

The methods described above have to date been applied in full to only one PF-derived food protein. For recombinant human lactoferrin expressed in *K. phaffii*, a Codex-based assessment combined a literature review of host allergenicity, full-length and 80-amino-acid sliding-window searches against AllergenOnline together with BLASTP, an allergen search for each of the 36 residual host proteins identified by mass spectrometry, an evaluation of the oligomannose N-glycans for cross-reactive carbohydrate determinants, and in vitro pepsin digestion compared side by side with human milk and bovine lactoferrin [62]. The recombinant protein showed no significant homology to known allergens and a digestibility profile comparable to human milk lactoferrin, and the authors concluded that neither serum IgE testing nor clinical testing was indicated [62]. A subsequent randomized, double-blind trial in 66 healthy adults measured serum anti-lactoferrin antibodies after 28 days of intake; no material change was observed in the recombinant groups, whereas the bovine lactoferrin control showed a 3.01-fold increase in anti-bovine-lactoferrin antibodies [82]. This trial did not enroll allergic individuals and measured total antibody rather than IgE, so it does not address IgE-mediated reactivity; nonetheless, it is the only human study of a PF-derived food protein reported to date. No comparable assessment has been published for the PF-derived milk and egg proteins that dominate the current market. β-Lactoglobulin accounts for most of the GRAS notifications listed in Table 1, and its allergenicity was assessed in the EU novel food evaluation of *T. reesei*-derived β-lactoglobulin [83]; however, the supporting allergenicity data were submitted as confidential annexes and are not available in the public literature. The evidence base therefore consists of one fully published case, concerning a protein with a native counterpart that is not a major food allergen, while the proteins that are themselves established allergens have been assessed only through non-public dossiers.

## 5. Future Perspectives

### 5.1. Process-Dependent Allergenicity and the Need for Process-Aware Evaluation

The allergenicity of PF-derived products is not fixed by the target sequence alone but is shaped jointly by the production process and subsequent food processing [34]. Because microbial hosts differ in their capacity to perform PTMs, proteins with identical amino acid sequences may differ in structure and immunological behavior depending on the host [22,24], and residual HCPs contribute to the immunochemical background of the final product [9]. Thermal and high-pressure processing, enzymatic hydrolysis, and glycation can further modify epitopes during formulation [56,58,63]. Because PF-derived products differ in the gene, host, medium, and processing conditions used, their allergenicity may vary from product to product [9]. Therefore, allergenicity cannot be judged at the level of the purified recombinant protein or the sequence but must be evaluated individually in the final formulated product.

Product-by-product evaluation carries a further implication. Equivalence is demonstrated on the batches submitted for assessment, whereas the material reaching consumers is the output of routine manufacture, in which glycan profiles and proteolytic clipping may vary with bioreactor conditions. A single demonstration of equivalence is therefore not sufficient on its own. The attributes on which the equivalence claim rests need to be defined as measurable specifications and monitored across batches, in the way that critical quality attributes are controlled for recombinant biologics.

Although no direct evidence currently indicates that PF-derived products differ substantially in allergenicity from their native homologs, this reflects the limited research conducted to date rather than demonstrated equivalence [34]. Regulators have accordingly identified allergenicity as a key data gap preventing conclusions on the safety of PF-derived products [34]. Closing this gap will require a systematic comparison of PF-derived and native proteins under identical processing conditions, combined with assessment frameworks that explicitly account for processing-induced changes in allergenicity. Moreover, no criteria or thresholds have yet been established for how much deviation in IgE binding, epitope structure, or digestive stability constitutes a significant loss of equivalence or an increase in allergenicity [18]. Establishing these thresholds is a prerequisite for making equivalence-based assessment rigorous and reproducible.

Comparison of allergenic risk across host strains and medium formulations is also needed. These choices are presently made on yield and cost alone. Determining whether one host renders the target protein more or less allergenic than another requires the same protein to be produced in different hosts and compared under matched conditions. Such data have not yet accumulated, so hosts and media cannot be ranked by allergenic risk. Generating the required evidence nonetheless appears tractable. The same target has been reported to be produced in several different hosts (Table 1), so further study using material already on the market could go some way towards establishing a controlled comparison under identical formulation and processing. Such a dataset would place host and medium selection on an immunological footing, to the benefit of both producers and regulators.

### 5.2. Predicting De Novo Sensitization and the Transition to Animal-Free Assessment

Allergenicity assessment distinguishes two objectives, viz., predicting cross-reactivity in already sensitized individuals and predicting de novo sensitization, and the induction of a new allergy in previously nonallergic individuals [84]. Cross-reactivity prediction based on sequence homology and serum IgE testing is comparatively mature, whereas de novo sensitization remains poorly understood, with no validated or internationally agreed predictive methods currently available [84,85]. Because PF targets are homologs of known allergens, cross-reactivity dominates; however, neoepitopes originating from N-terminal extensions or non-native modifications may introduce sensitizing determinants that are absent from the native protein [86]. Such risks are not captured by cross-reactivity methods; hence, the structural deviations of PF-derived products in fact elevate the need for de novo sensitization prediction.

Assessment of sensitizing potential has depended, partially, on animal models, which do not completely reproduce human immune responses and whose outcomes vary with strain, allergen source, adjuvant, and sensitization route [87]. Therefore, human-based alternatives are being developed, including epithelial–immune cell cocultures, organoids, and gut-on-chip platforms [88]. An in vitro human mucosal immune model reproducing sensitization from intestinal epithelial cell activation through dendritic cell and naive T-cell priming to IgE isotype switching and mast cell degranulation has been demonstrated using ovalbumin [89]. Artificial intelligence(AI)-driven in silico approaches are also promising; however, standardized datasets and external validation remain limiting [90]. Hence, it is necessary to develop and validate human-relevant animal-free methods that also capture the de novo sensitization risk posed by structural deviations in PF-derived products.

### 5.3. Definitional Ambiguity of PF and Its Regulatory Implications

The scope of safety assessment required for PF-derived proteins depends on how the product is classified, yet the term itself lacks a harmonized definition. PF was introduced to distinguish the genetic modification of a microbial host for targeted molecule production from traditional and biomass fermentation [91]; however, this manipulation encompasses both the introduction of foreign genes and genome editing with tools such as CRISPR-Cas, and a formal categorization of engineered microorganisms remains to be established [92]. If the mode of genetic manipulation is incorporated into the definition, almost identical products may follow different regulatory routes depending on the technique used.

This ambiguity has practical consequences because it determines the data required for allergenicity assessment. When a genetically modified microorganism carrying foreign genes is used, the route falls under either GMO legislation or the novel food regulation depending on whether viable cells or recombinant DNA persist in the final product, and molecular characterization of the production strain is required in either case [34]. However, for microorganisms modified only by genome editing, classification remains unresolved across jurisdictions, because existing GMO regulations were developed for established modification methods rather than genome editing. Therefore, establishing allergenicity assessment procedures for PF-derived proteins requires a clear definition of the scope of PF, together with a systematic mapping of which assessment requirements apply to each mode of genetic manipulation.

## 6. Conclusions

Because PF-derived proteins are homologs of proteins already established as allergens, such as those of milk and egg white, their assessment is based on equivalence to the native counterpart rather than on the identification of a novel hazard. This equivalence is not self-evident. Host-dependent PTMs, incomplete signal peptide cleavage, non-native disulfide patterns, and residual HCPs cause PF-derived proteins to deviate subtly from their native counterparts. These deviations both alter epitopes directly and regulate how the protein responds to subsequent processing, during which heat, high pressure, enzymatic hydrolysis, and glycation can destroy, mask, or expose epitopes and generate neoepitopes. Because such deviations differ with the gene, host, and process used, allergenicity cannot be established at the level of the target sequence or the purified protein; it must be judged product by product in the final formulated food, after realistic processing, in direct comparison with the native counterpart.

The established tiered scheme remains a valid framework for this comparison, although each tier requires reinterpretation for PF-derived proteins, and molecular endpoints alone are inadequate because changes in IgE binding do not necessarily correspond to changes in clinical reactivity. A comparison with food enzymes clarifies what carries over and what requires adaptation. The control of residual host cell proteins and medium-derived allergens transfers directly, since these are common to food proteins of fermentation origin. The function of sequence homology analysis, by contrast, requires adjustment. In food enzymes, this tier identifies unknown hazards, whereas for PF-derived proteins the target is essentially identical to the database entry and a complete match is returned trivially. The informative content therefore lies in the regions where the recombinant sequence departs from the native one. Digestibility and IgE-binding tests likewise shift in what they establish. They ask not whether the protein is an allergen but whether it behaves as its native homolog does, which calls for comparison against a counterpart assayed in parallel rather than an absolute judgment. Three further requirements arise that food enzyme assessment does not address. These are (i) evaluation in the final formulated product rather than the purified protein; (ii) thresholds for judging when equivalence has been lost; and (iii) control of batch-to-batch consistency in the attributes that are immunochemically relevant. Meeting these requirements would establish an equivalence-based allergenicity assessment suited to PF-derived proteins without replacing the existing framework. Direct comparative data generated under identical processing conditions and a harmonized definition and regulatory classification of PF are needed alongside this.

## Figures and Tables

**Table 1:** Recombinant milk and egg proteins produced by precision fermentation that have received FDA “no questions” letters under the GRAS notification program, as of 1 September 2026.

GRN	Substance	Production Organism/Strain	FDA Response
863	β-Lactoglobulin	*Trichoderma reesei* “QM6a-PD1”	25 March 2020
967	Soluble egg-white protein, predominantly recombinant ovomucoid	*Komagataella phaffii* GSD-1209	9 September 2021
1056	β-Lactoglobulin	*K. phaffii* “yRMK-66”	15 February 2023
1104	Egg-white protein, predominantly recombinant ovalbumin	*K. phaffii* ATCC GSD-1235	17 October 2023
1145	β-Lactoglobulin	*Aspergillus oryzae* “Ao_st0002”	18 December 2023
1200	β-Lactoglobulin	*K. phaffii* “VIPLA”	28 February 2025
1219	Recombinant bovine lactoferrin isolate	*K. phaffii* “M020”	7 May 2025
1241	β-Lactoglobulin	*A. oryzae* (strain not specified in the inventory)	16 September 2025
1247	β-Lactoglobulin, expressed from a bovine gene	*Kluyveromyces lactis* CCTCC M20241460	19 September 2025
1249	Ovalbumin	*T. reesei* ATCC 13631	16 September 2025
1284	Recombinant bovine lactoferrin isolate	*K. phaffii* “Ppas_337”	25 March 2026

Data were retrieved from the US FDA GRAS Notice Inventory (https://www.hfpappexternal.fda.gov/scripts/fdcc/index.cfm?set=GRASNotices), accessed on 30 July 2026.

**Table 2:** Comparison of the host groups used to produce PF food proteins.

	Bacteria	Yeasts	Filamentous Fungi
Representative hosts	*E. coli*, *B. subtilis*	*S. cerevisiae*, *K. phaffii*	*T. reesei*, *A. niger*
N-glycosylation imparted to the target	None, as eukaryotic glycosylation machinery is absent	High-mannose N-glycans where the target sequence carries N-glycosylation sites, in contrast to the complex type found in mammals	High-mannose N-glycans, with Man5 reported for proteins produced in *T. reesei*
Host-derived material persisting in the product	Residual HCPs and host-derived polysaccharides, with residual endotoxin where the host is Gram-negative	Residual HCPs and host-derived polysaccharides	Residual HCPs and host-derived polysaccharides, together with mycotoxins where the strain retains the capacity to produce them
Deviation imparted to the target during expression and secretion	Folding heterogeneity, with minor differently folded isomers detected in the recombinant protein	N-terminal extension from incomplete cleavage of the secretion leader, absence of native N-terminal acetylation, and trimming of termini by host proteases	Fragmentation by host proteases during secretion

## Data Availability

No new data were created or analyzed in this study. Data sharing is not applicable to this article.

## Abbreviations

The following abbreviations are used in this manuscript:

AGEs	advanced glycation end products
AI	artificial intelligence
AOX1	alcohol oxidase 1
BLAST	basic local alignment search tool
BLASTP	protein basic local alignment search tool
CRISPR-Cas	clustered regularly interspaced short palindromic repeats and CRISPR-associated protein
EFSA	European Food Safety Authority
ELISA	enzyme-linked immunosorbent assay
EU	European Union
FDA	Food and Drug Administration
GMO	genetically modified organism
GRAS	generally recognized as safe
GRN	GRAS notice number
HCPs	host cell proteins
HPP	high-pressure processing
IgE	immunoglobulin E
IgG	immunoglobulin G
IUIS	International Union of Immunological Societies
NMR	nuclear magnetic resonance
PF	precision fermentation
PTMs	post-translational modifications
WHO	World Health Organization
